# An Effective Deep Learning Model for Health Monitoring and Detection of COVID-19 Infected Patients: An End-to-End Solution

**DOI:** 10.1155/2022/7126259

**Published:** 2022-08-11

**Authors:** Vidyadevi G. Biradar, Mejdal A. Alqahtani, H. C Nagaraj, Emad A. Ahmed, Vikas Tripathi, Miguel Botto-Tobar, Henry Kwame Atiglah

**Affiliations:** ^1^Department of Information Science and Engineering, Nitte Meenakshi Institute of Technology, Bangalore, India; ^2^Department of Industrial Engineering, King Saud University, Riyadh, Saudi Arabia; ^3^Nitte Meenakshi Institute of Technology, Bangalore, India; ^4^Department of Computer Science, Faculty of Computers and Information, South Valley University, Qena, Egypt; ^5^Department of Computer Science and Engineering, Graphic Era Deemed to Be University, Dehradun, Uttarakhand, India; ^6^Eindhoven University of Technology, Eindhoven, Netherlands; ^7^Research Group in Artificial Intelligence and Information Technology, University of Guayaquil, Guayaquil, Ecuador; ^8^Department of Electrical and Electronics Engineering, Tamale Technical University, Ghana, Ghana

## Abstract

The COVID-19 infection is the greatest danger to humankind right now because of the devastation it causes to the lives of its victims. It is important that infected people be tested in a timely manner in order to halt the spread of the disease. Physical approaches are time-consuming, expensive, and tedious. As a result, there is a pressing need for a cost-effective and efficient automated tool. A convolutional neural network is presented in this paper for analysing X-ray pictures of patients' chests. For the analysis of COVID-19 infections, this study investigates the most suitable pretrained deep learning models, which can be integrated with mobile or online apps and support the mobility of diagnostic instruments in the form of a portable tool. Patients can use the smartphone app to find the nearest healthcare testing facility, book an appointment, and get instantaneous results, while healthcare professionals can keep track of the details thanks to the web and mobile applications built for this study. Medical practitioners can apply the COVID-19 detection model for chest frontal X-ray pictures with ease. A user-friendly interface is created to make our end-to-end solution paradigm work. Based on the data, it appears that the model could be useful in the real world.

## 1. Introduction

The outbreak of Coronavirus Disease 2019 (COVID-19) is a threat to mankind that has halted the entire world and forced everybody to isolate and quarantine to save themselves. It is extremely important to test and investigate positive COVID-19 infected cases as soon as possible to prevent this contagious disease. Chest radiological imaging such as X-rays has a crucial role in the timely detection, diagnosis, and treatment plan of this disease. Chest X-rays have been implemented majorly by almost all healthcare facilities nowadays[1].

COVID-19 is caused by the SARS-CoV-2 virus infection, which is spread through coughing, sneezing, and so on. Some patients have long-term breathing problems, heart disease, severe kidney problems, and so on; as a result, it is life-threatening and should be addressed as soon as possible [2].

The COVID-19 infection is life-threatening and there is a scarcity of diagnosis facilities in rural areas. The pandemic has put a heavy workload on radiologists, who need support from automated tools to ensure that infected patients seek treatment attention. The deep learning models have proven their abilities in medical image analysis on par with experts. Therefore, this research work proposes a deep learning model for COVID-19 detection that is built for web and mobile-enabled applications. With this tool, medical staff can upload the chest X-ray of the patient and fetch the results of the analysis [3]. The application developed in this paper also provides the facility to monitor the health parameters of the patient.

It is imperative that infected people must be tested as soon as possible to stop the spread of the disease and defeat it. Physical approaches are time-consuming, expensive, and tedious. Because of this, there is a pressing need for a cost-effective and efficient automated tool. In this work, a deep learning method for analysing X-ray pictures of patients' chests is given [4]. This study investigates the most suitable pretrained deep learning models that can be integrated with mobile or online apps and support the mobility of diagnostic instruments in the form of a portable tool. Patients can use the smartphone app to find the nearest testing healthcare facility, book an appointment, and get instantaneous results, while healthcare personnel can keep track of the details thanks to the web and mobile applications built into this study. For frontal chest X-ray images, the COVID-19 detection model can be used with simplicity by medical practitioners. A user-friendly interface is created to make our end-to-end solution paradigm work. Based on the findings, it appears that the model can be put to good use as a real-world tool [5].

RT-PCR tests are effective for diagnosing infection; however, they fail to detect infection in the very early stages of infection. Also, these tests are tedious and time-consuming. The COVID-19 infection analysis can be carried out better by combining pathological RT-PCR testing with inferences from chest X-rays. According to the expert study, the opacities pattern in the lungs-infected patient's chest X-ray gives vital information about the type of pneumonia infection and its type, which can be identified using AI techniques for image analysis. These AI techniques are very useful in reducing the workload of radiologists with satisfactory levels of the result.

Images from chest X-rays and CT scans are used for detailed analysis, and infection analysis is performed automatically. The radiologists manually inspect the images wherever AT tools do not exist. AI tools aid in early diagnosis and prevention of infection spread, as well as a correct treatment plan for the afflicted patient.

Radiographic images are generally used as the first stage of diagnosis to test and evaluate a patient's X-ray to detect if the person is showing COVID-19 symptoms or is suspected to be infected by the contagious disease. Numerous studies have reported that there is a very close relation between lung abnormalities on chest X-rays and the bleakness of the disease [6]. It has shown an increase in the intensity of lung opacities that are associated with clinical admittance, an exponentially high number of ICU admissions, and, in worse cases, death. Chest radiology is a vital part of assessing and analysing chest X-rays to diagnose COVID-19 in an affected patient.

Chest CT scans are effective in COVID-19 detection. However, they can be used for the second-level analysis of infection spread as CT scans involve comparatively lengthy procedures. Often, chest X-rays are useful due to their low cost and easy availability in hospitals.

Detection of COVID-19 happens through a reverse-transcription-polymerase-chain reaction (RT-PCR) test. It results in a satisfactory diagnosis when combined with the X-rays of the patient. According to researchers and experts, chest X-rays are used to examine and detect opacities in the lungs, which infers COVID-19 detection. Radiologists are in high demand during the pandemic because they will have to test and report on a large number of patients worldwide [7]. The humongous workload placed on radiologists as a result of their growing number would be a factor impacting the test's accuracy.

The COVID-19 infection is confirmed with an RT-PCR test according to conventional clinical methods. However, it is found that its sensitivity level is not sufficient to detect infection in the early stages. The chest CT-images and chest X-ray images demonstrate texture patterns that correlate to the existence of infection. Deep learning techniques have recently seen a great deal of success in the medical field since they are great at analysing image texture patterns. These AI-based methods reduce the workload and add to the confidence of expert radiologists in the ambiguous situation [8].

Considering this notion, Artificial Intelligence (AI) provides support in analysing and testing the uploaded chest X-rays, which in turn reduces the humongous workload on radiologists. As mentioned previously, because of the increasingly small number of radiologists, there is a huge need for the development of tools for automating the diagnosis of chest X-ray images for the detection of patients affected by corona infection. Medical doctors are facing the difficulties of COVID-19 diagnosis, which is supported by an artificial intelligence system that accelerates the observation of infection by inspecting chest X-rays. This is accomplished by detecting and diagnosing opacities in the lungs that are cross-related to COVID-19 [9].

The web and mobile applications for COVID -19 diagnosis provide facilities to the rural areas where hospitals are equipped with minimal health monitoring support. Therefore, these applications enable access to diagnostic services by any remotely located person. Hospitals in rural areas need automated tools for COVID-19 diagnosis as there is a scarcity of radiologists. An automated tool that is cost-effective and worthy to cater to the requirements of the community in a broader way. Hospitals in rural areas need automated tools for COVID-19 diagnosis as there is a scarcity of radiologists. An automated tool is cost-effective enough to cater to the requirements of the community in a broader way.

The system abetted tools that are mainly created and designed using Convolutional Neural Networks (CNN) pretrained models have been extremely accurate in determining and locating the pneumonic infections and lung opacities from the input of chest X-ray images. There are multiple pretrained models available that can be used to determine pneumonic infections and lung opacities from the data input, and a few of them have far exceeded experts' opinions. In this paper, we will discuss the best pretrained model to utilize out of the lot to outperform the others and simultaneously give accurate results [10].

Radiographic inspections are extremely good at predicting and assessing the course of COVID-19. Experts or radiologists can assess the presence of COVID-19 in a patient by noticing patterns in chest X-ray patterns. These patterns are ground-glass opacities, crazy paving, Vascular dilation, and so on. In COVID-19-infected patients, consolidation opacities and reticular interstitial thickening were the most common issues. As a result, the deep learning and machine learning communities have been constantly expanding their reach and exploring along the lines of diagnosing COVID-19 infection and testing the feasibility of the deep-learning paradigm in this phase [11].

This research work describes a survey on the application of convolutional neural networks for the diagnosis of coronavirus infection through chest X-rays and an automated way in which patients and doctors can detect COVID-infected patients and keep track of all the data.

The pretrained deep learning models that we will be implementing are complex, and since the heavyweight AI models need to also be able to run on commodity hardware, there is demand for lightweight alternatives having minimal accuracy trade-offs. Several studies are trying to incorporate different transfer learning techniques on various pretrained models to evaluate the differences in accuracies. This is because an actual production level model has not been deployed enough as comprehensive datasets are hard to formulate. However, the situation is changing, and with larger datasets being compiled and updated frequently, online learning can be implemented to have a fast and easy-to-update Deep Transfer Learning Model [12].

The contents of this paper give an insight into how efficient and suitable pretrained models are in detecting COVID-19 infections. The models that we have segregated and used are SqueezeNet [13–15], DenseNet [16], and ResNet [17]. The contribution of research work includes a selection of the best pretrained models, their applicability of augmentation approaches for COVID-19 positive image samples, and enumerates a list of possible datasets with necessary details that are utilized in research exploration on COVID-19 detection using X-ray images of the chest.

This work presentation is organized into various sections.  Section 2 elaborates on related work, Section 3 explains the methodology, Section 4 explains the research direction and challenges, Section 5 concludes the research done on this project, and Section 6 contains all the references.

## 2. Related Work

Deep learning is based on the fundamentals of machine learning that follows the concept of neural networks to solve numerous problems, which are inspired by the functioning of the human brain comprises an exponential number of neurons that are connected to each other and communicate through electrical signals.

In a paper [18], Deep Learning is an approach for automated feature extraction to be dealt with. Convolutional neural network models are exploited in feature extraction in image processing applications. In convolutional neural networks, more primitive image features are detected by the initial layers, whereas middle layers and later layers detect features that are more significant to images.

Therefore, through the reuse of preacquired knowledge of pretrained CNN models, a novel challenge is being solved. The performance of deep learning techniques is constrained by the size of the image dataset.

In a related work by Boran Sekeroglu, Ilker Ozsahin [19, 20], chest X-rays are used because the chest X-ray is the most commonly performed examination for the diagnosis of a patient. An X-ray/radiographic image is a noninvasive medical test that helps doctors to test, diagnose, and treat medical conditions depending on the impact.

In the situation of the corona pandemic chest, X-ray allows very fast triaging, as X-ray equipment is existing in health care centers. The procedure of acquiring an X-ray also occurs in a confined space so there is minimal risk of transmission of COVID. The application of CNN models is proven to be accurate and efficient in COVID-19 diagnosis, thus reducing inaccuracies introduced by the workload of radiologists [21].

The pretrained models SqueezeNet [14], ResNet18, and its variants through transfer learning are explored through analysis of chest CT scans. Imbalanced datasets are dealt with using wavelets in combination with CNNs and the performance of the ResNet18 is found to be satisfactory [22]. The authors in [23] have presented a combinational model with RNN and CNN called the ProgNet model for observing the progression of infection in temporal images through time series; the model results were 92% accurate in detecting the infection progression. The corona infection is analyzed using multiple models—the first model is designed using CNN-based model, the second is developed using pretrained models DenseNet121 and ResNet50 with the help of transfer learning methods, and the third model COVID-CXNet is designed with pretrained CheXNet; it is found that COVID-CXNet outperformed the other two [24]. An ensemble model using ResNet50, VGG-16, and GoogLeNet pretrained models is implemented for COVID-19 feature extraction, and classification is performed using support vector classification (SVM); this model gives an accuracy of 95.60%, which is found to improve as compared to individual models [25].

## 3. Methodology

### 3.1. COVID-19 Binary Classifier

The frontal lung X-rays of coronavirus-positive people can be differentiated from healthy people fairly accurately by radiologists. We have used the COVID-XRay-5K dataset [26], which has been created using the COVID-Chest X-ray image dataset [27], for COVID-19 X-ray images and the ChexPert image dataset [28], for negative COVID samples. This dataset has been filtered by an expert radiologist and only those who were selected to have a clear sign of COVID-19 were kept, along with the posterior-anterior images. Non-COVID images are uniformly sampled from the ChexPert dataset. As the samples of COVID-19 images are fewer than non-COVID images, data augmentation along with oversampling is performed on the COVID-Chest X-ray dataset is used to have a balanced training set.

**Figure 1:**
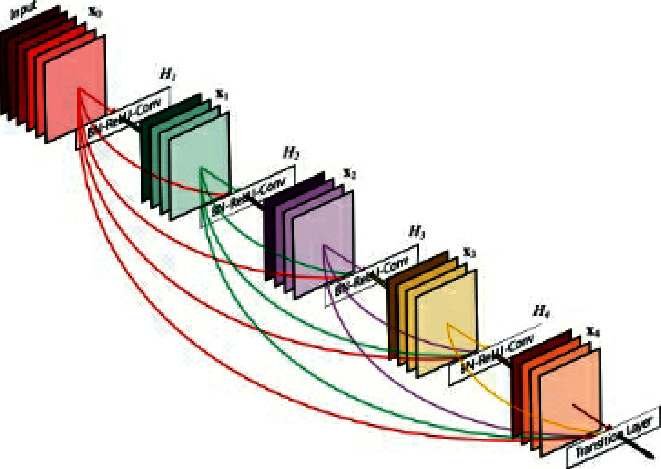
DenseNet architecture.

We chose to train multiple model types for comparison of training times and accuracy metrics, leaning towards models that take the least amount of training time and computational resources. The chosen models were pretrained versions of DenseNet121 [16], ResNet18 [17], and SqueezeNet [13, 14, 29]. The architecture of DenseNet is shown in Figure 1. DenseNet [16] can be trained with relatively fewer parameters than other models, and since each layer takes the reduced feature maps of the previous layer as inputs, feature propagation is strengthened.

**Figure 2:**
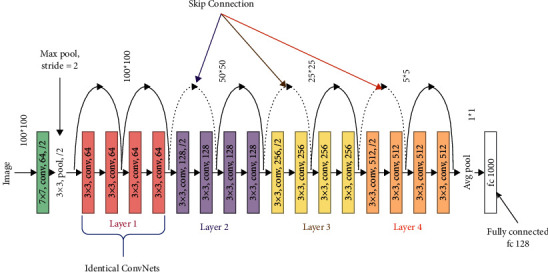
ResNet-18 architecture.

The theme of ResNet [17] is introducing a so-called “identity-shortcut-connection” that skips the suitable number of layers as shown in Figure 2, which helps solve the vanishing gradient issue as a new shortcut is created for the gradient to flow through. A different way in which these connections can allow the deep learning model to learn functions that can ensure the higher layer would be performing at least as good as the lower layer performed; this is how it can help.

**Figure 3:**
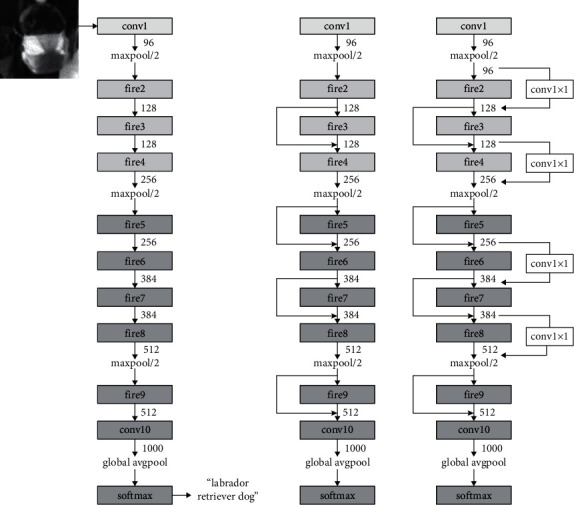
SqueezeNet architecture.

SqueezeNet [13, 14, 29] is a CNN network shown in Figure 3 that has faster training times and tiny size. Its main idea is to use point-wise filters instead of 3 × 3 filters, reducing computation to 1/9th of the original network. By using 1 × 1 filters as bottleneck layer, depth computation of 3 × 3 filters is reduced. This leads to smaller Convolutional Neural Networks, which can be easily deployed to devices with limited hardware and memory capacities. Smaller CNN also needs less bandwidth while updating and deploying.

The architectures of ResNet, DenseNet, and SqueezeNet models are lightweight; therefore, these pretrained models are suitable for web and mobile applications. The pretrained models such as VGG16 and GoogLeNet are comprised of very-deep-layered CNNs; thus accounting for complex architectures, these are not suitable for mobile applications as they demand lightweight models.

The overall approach is as follows:

  (1) Identify and segregate COVID-19 positive and negative images and split the images for training, testing, and validation purpose.
  (2) Resize all images to a uniform size; a size of 224 × 224 is selected.
  (3) Train DenseNet121 [16], ResNet18 [17], and SqueezeNet [13, 15, 29] through the training set images to execute loss minimization using the test set image dataset. Among each model type, choose the model with the best validation accuracy. Adjust parameters and reach a final model and compare the trained models.
  (4) Test the trained models on the validation image dataset and compute metrics.

### 3.2. Dataset

**Figure 4:**
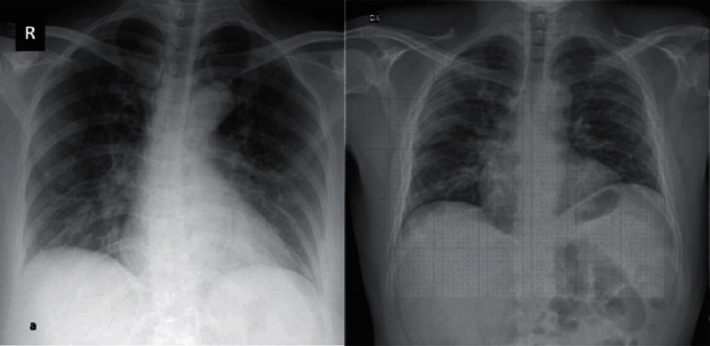
Positive images.

**Figure 5:**
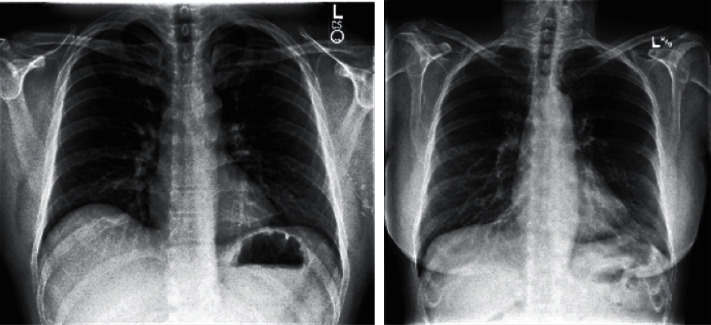
Negative images.

This research work uses COVID-X-ray-5K dataset [26], which is the integration of datasets COVID-Chest-X-ray dataset [27] and ChexPert dataset [28]. The resulting dataset was authenticated by radiologists after the removal of ambiguous images about positive and negative COVID-19 cases. The sample images representing positive and negative cases are shown in Figures 4 and 5, respectively. The distribution of image samples indicates that the dataset is imbalanced; therefore, image augmentation is applied during the training of the model.

It is difficult to match image characteristics of chest X-ray images, which are collected from different modalities. Therefore, image augmentation strategies are explored to nullify the negative effect of the imbalanced dataset.

### 3.3. Preprocessing

Image augmentation strategies, translation, flipping and contrast change, and so on, are implemented to enhance the dataset to solve both the issues of limited dataset and the imbalanced dataset. The image normalization was performed and then resized into 224 × 224. The training data have 234 images, of which 84 are class 0 and 150 are class 1 as tabulated in Table 1. The testing and validation set each has 50 images of class 0 and 100 images of class 1. Data transforms to randomize training are then applied while loading the images and the dataset is initialized.

Data for training, test, and validation:

The selected images are normalized and then resized into 224 × 224. The training data has 234 images, of which 84 are class 0 and 150 are class 1. The testing and validation sets each has 50 images of class 0 and 100 images of class 1. The deep learning networks need a huge amount of image samples; therefore, augmentation is applied to improve the dataset. The augmentation techniques applied include transformations, rotation, and flipping operations. The data transforms to randomize training are then applied while loading the images and the dataset is initialized.

The model, when trained on a dataset that contains the imbalanced distribution of types of samples, positive and negative, models will be overfitted and hence accuracy of classification will deteriorate.

#### 3.3.1. Tools

We have used a physical machine (Intel I7 8750H, NVIDIA 1050 TI 4 GB GDDR5 VRAM, 16 GB RAM), Python 3.7, and PyTorch 1.7.0.

#### 3.3.2. Training

Multiple model versions were trained and tweaked, and parameters are chosen as given in Table 2. All models are trained for 100 epochs, SGD optimizer and loss function as Cross-Entropy, learning rate value 0.0001, and momentum of 0.9. The time taken for training is 8 s/epoch for SqueezeNet [13, 14], 8.4 s/epoch for ResNet, and 10.4 s/epoch for DenseNet. The performance is evaluated on classification accuracy, sensitivity, and *F*1 score. Table 2 defines the different model parameters used for training.

The training of ResNet, DenseNet, and SqueezeNet are perfumed through a transfer learning paradigm, where the lower layers of the models are kept frozen as the primitive features of the images in general and therefore the weights in the lower change remain the same to the larger extent. The higher-level layers are retrained to learn the features from COVID-19 images more precisely.

## 4. End-to-End Solution

After a satisfactory model was selected and trained, an end-to-end solution that can be used by patients and testing centres was implemented. The entire workflow revolves around the COVID-19 binary classifier and makes the fullest use of its capabilities, which are demonstrated as follows:

  (1) The patient can register on the mobile application and search for the nearest COVID-19 testing centres. A testing centre is chosen, and a test is booked.
  (2) A web application is deployed at testing centres for diagnosis of COVID-19 infection from chest X-ray of a patient.
  (3) The real-time diagnosis of infection is carried out by uploading the chest X-ray to the web application dashboard.
  (4) The deep learning model running in the cloud analyses the X-ray and indicates results.
  (5) Implementation.

Even with all the data and pretrained models available, to gain maximum accuracy, it was needed to train the Squeeze Net [29] CNN model on chest X-ray images. However, for testing the output of the model, it was needed to create an end-to-end interface for connectivity with users and proper testing with different types of users.

To overcome the situation, an architecture of working applications with servers was designed that can be accessed from anywhere in the cloud.

**Figure 6:**
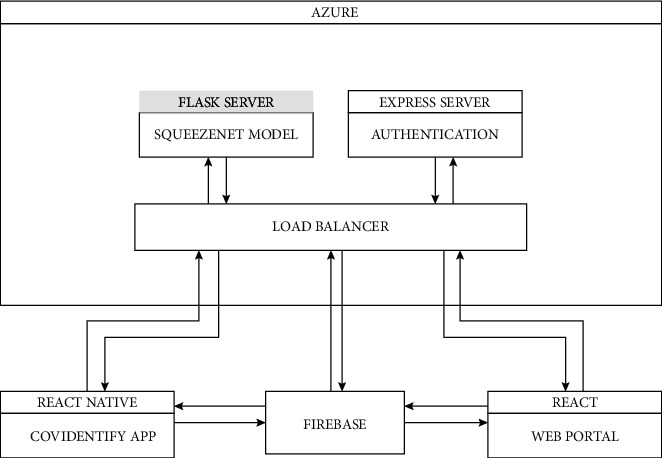
Architecture design.

Figure 6 represents the architecture design of the working application with the SqueezeNet [29] model trained on chest X-ray images. The infrastructure consists of seven elements:

1. Azure (cloud service provider)
2. Flask server
3. Express server
4. CovIdentify mobile application
5. Load balancer
6. Firebase
7. Web portal

### 4.1. Azure

Azure is the cloud-computing platform by Microsoft. It provides a heterogeneous range of cloud services like computing, analytics, storage solutions, and networking solutions. The role of Azure in the architecture is hosting the servers on the cloud so that they can be accessed anywhere over the Internet by end-users at any point in time.

In the work, Azure is used for hosting the Flask server and the Express server so that they can be accessible by the end-to-end applications like mobile applications and web applications from anywhere over the Internet.

### 4.2. Flask Server

Flask is a web framework written in Python used for creating back-end servers without using any level concept. The role of the Flask server in our architecture is to host the trained SqueezeNet on Azure for connectivity of the model to other services for taking input of user's chest X-ray images and real-time producing the results that can be accessed by other services to notify the end-users.

### 4.3. Express Server

Express is a web-application framework developed in JavaScript for creating back-end servers same as the Flask server.

The express server in our infrastructure holds the core logic of verifying and validating the end-user. Since the medical data are highly sensitive and cannot be exposed to the outer world, it is necessary to implement an authentication service to keep the X-ray images and user data accessible only by the users or administrative authority.

Apart from security purposes, the Express server is also responsible for handling the upload/download of the X-ray images provided by the user and storing it on the file system of the server so that they can be accessed by the SqueezeNet model to classify the images as Covid negative and positive.

### 4.4. CovIdentify Mobile Application

The most important aspect of the overall application is to help the users seamlessly access the services and help them to identify COVID positively or negatively.

The mobile application provides an interface to the users with a beautiful user experience helping to connect to the services.

The mobile application is built on React-Native, an open-source hybrid mobile application development framework backed by Facebook.

The features of the CovIdentify app are as follows:

  (1)An interface for end-user is provided to book slots for the available testing centres that provide X-ray facilities
  (2)It provides the history of all the past tests as well as the status of the pending test report of the user

### 4.5. Load Balancer

Since the servers are distributed and loosely coupled, it was necessary to create a single access point to interact with the back-end server for the mobile application and the web portal.

Load balancer acts as a single-entry point for all the services that redirect the requests to the particular service that needs to be accessed at a particular time.

### 4.6. Firebase

Firebase is created by Google. It provides the authentication logic for seamless authentication of users using e-mail, social-media accounts, and others of the particular user.

For hassle-free login, Firebase is used in the infrastructure.

### 4.7. Web Portal

The web portal is an end-user interface used by the hospital authority. It is developed on React, which is a JavaScript library developed by Facebook for creating single-page applications to be accessed by web browsers.

The web portal provides features like

  (1) Accessing the user's profile
  (2) Approving/rejecting user's request for booking slot in that particular hospital
  (3) Uploading of user's X-ray to the server to get the results

### 4.8. Application Screenshot

#### 4.8.1. CovIdentify Android Application

**Figure 7:**
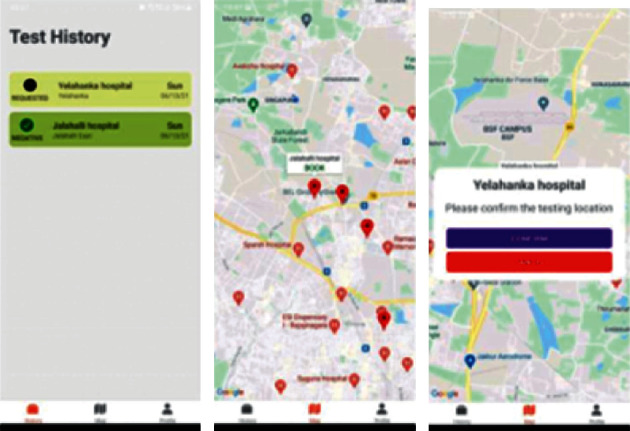
(a) Test history. (b) Test centers on map. (c) Booking a test center.

In Figure 7, the three images represent the basic workflow of the CovIdentify mobile application.  Figure 7(a) is the Test Screen which shows the follows:

- The pending request for a slot booked in a hospital
- The test history of previously done tests

Figure 7(b) is the image of the Map screen, which shows the available hospitals that are the testing centres and can be booked.

Figure 7(c) shows the confirmation pop-up of booking a hospital appearing when a user tries to book a slot in a hospital. It consists of two options that are “Confirm” and “Cancel.” Pressing the “Confirm” button will send a hospital slot booking request and the status of booking the test center will be displayed on the “Test History” screen (Figure 7(a)).

#### 4.8.2. Web Portal

**Figure 8:**
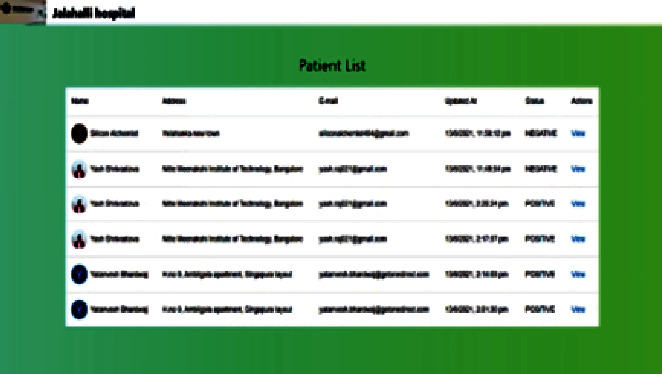
Web portal patient history screen.

As the functionality of the Web portal is mentioned earlier, Figure 8 shows the history of the patients that booked the particular hospital for testing.

The screen displays different attributes like the following:

- Name: displays the name of the patient
- Address: displays the address of the patient
- E-mail: displays the e-mail address of the patient
- Updated at: displays the date and time of the patient's request status
- Status: displays the status of whether the patient is COVID negative or positive
- Action: allows the portal user to submit the X-ray report to detect the infection in the chest of the patient by navigating to the X-ray upload screen

**Figure 9:**
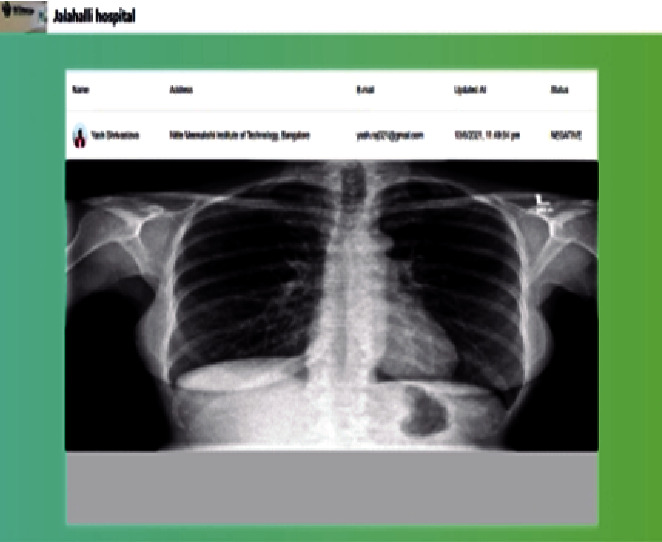
X-ray upload screen.

Figure 9 represents an X-ray uploading screen where the portal user at a hospital can upload the X-ray image of the patient's chest to obtain the result of an infection in the lungs.

## 5. Results and Analysis

**Figure 10:**
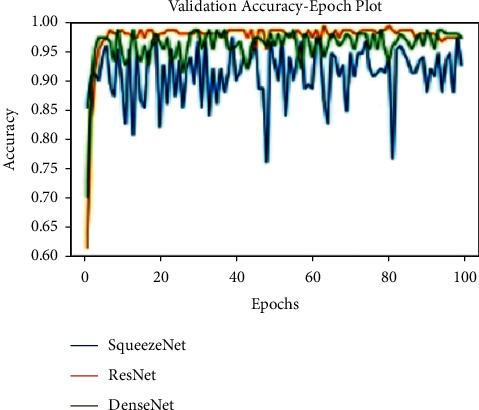
Validation accuracy plot.

**Figure 11:**
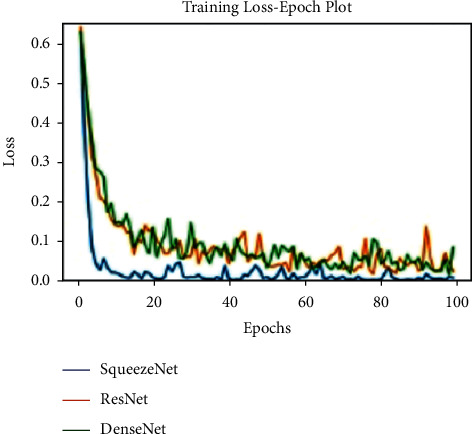
Training loss plot.

**Figure 12:**
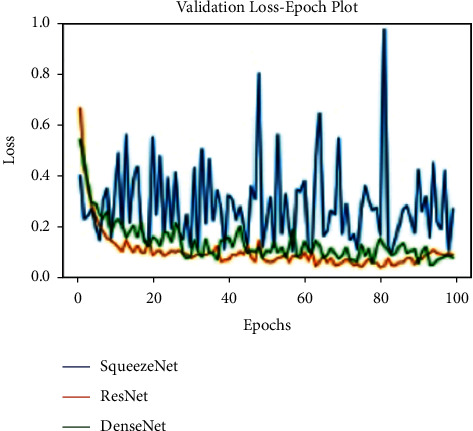
Validation loss plot.

All models are trained for 100 epochs, SGD optimizer. Loss function Cross Entropy with a learning rate value of 0.0001 and model momentum of 0.9. The time taken for training is 8 s/epoch for SqueezeNet, 8.4 s/epoch for ResNet, and 10.4 s/epoch for DenseNet. Figures 10, 11, and 12 show the performance of different models and DenseNet and ResNet give the most consistent performance; SqueezeNet due to its simplicity is sometimes not able to generalize accurately.

Table 3 represents the count of true-positive and true-negative classification values of COVID +ve and COVID –ve images. DenseNet shows impressive metrics but when compared to SqueezeNet, which is the relatively simpler model, SqueezeNet still performs adequately. The model sizes are 2.9 MB for SqueezeNet, 43.7 MB for ResNet, and 27.8 MB for DenseNet. In this regard and time to predict a single image, SqueezeNet can perform very fast predictions under lower-end hardware conditions. Under these considerations, SqueezeNet is chosen for final model deployment.

**Figure 13:**
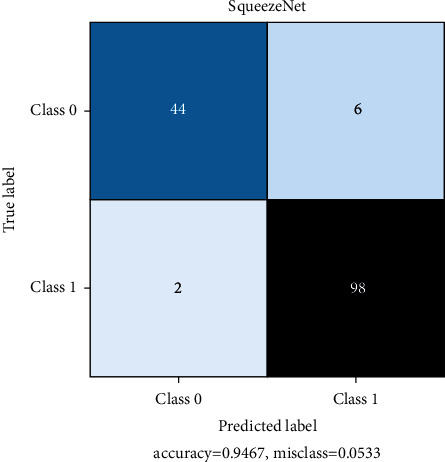
SqueezeNet confusion matrix.

**Figure 14:**
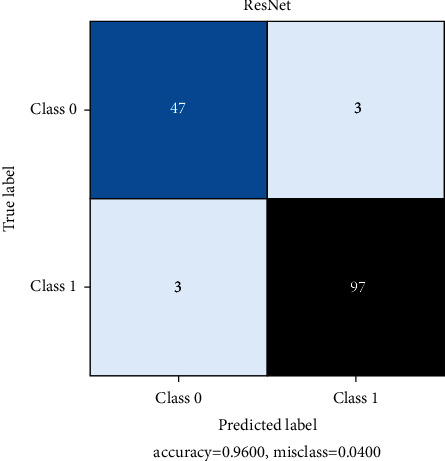
ResNet confusion matrix.

**Figure 15:**
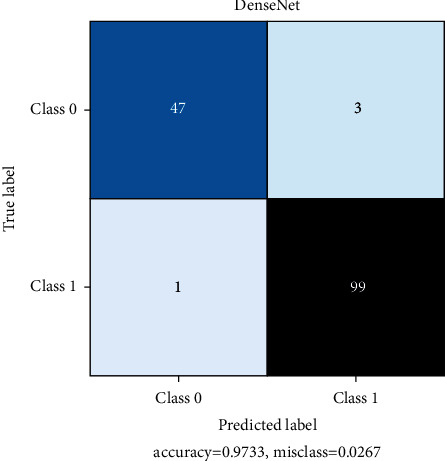
DenseNet confusion matrix.

Figures 13, 14, and 15 represent the confusion matrix for SqueezeNet, ResNet, and DenseNet. They provide the True Positive, True Negative, False Positive, and False Negative values.

Table 4 compares the time of prediction time for single and multiple (200) images for different CNN models trained on the same dataset. From the table, we can conclude that the prediction time of SqueezeNet [29] is faster than the other two CNN models we used.

Table 5 represents the different prediction scores on different metrics for all the CNN models trained on the same chest X-ray image datasets.

After considering and comparing all the metrics, we understand that SqueezeNet [29] can be used for the classification of the disease as its performance is very remarkable as the output can be obtained in a very small amount of time with good accuracy. The model's performance was deemed satisfactory by radiologists from the local hospital.

The paper concentrates on the classification model, which is a back-end technology in our product. However, to facilitate the use of the classification model, two front-end applications are designed for the end-user to access the model and help them detect the coronavirus infection by registering the information of the patient.

The project is a working product, putting together the front-end and back-end with the connectivity in between, and can be used by any individual to detect the infection.

## 6. Conclusion

The research work presents an automatic tool for COVID-19 diagnosis deployed on a mobile application to help patients access diagnosis facilities. This paper presents a model designed for COVID-19 infection detection in lungs using pretrained convolutional neural networks based on ResNet18, DenseNet121, and SqueezeNet pretrained models for web and mobile-enabled applications. The contributions of the paper include the design of user-friendly web and mobile application interfaces for uploading chest X-ray images and monitoring the general health parameters of the patient. The features of the applications help users or doctors to get a COVID-19 testing centre and enable them to fetch instantaneous results using chest X-rays. The model implemented is suitable for low-end workstations and less commodity hardware. For future endeavors, the model can be updated from a binary to a multiclass classifier for general-purpose lung infection identification [30].

## Figures and Tables

**Table 1:** Number of images.

Datasets	Corona +ve	Corona −ve	Total
Training	100	150	250
Validation	50	100	150
Testing	50	100	150

**Table 2:** Model parameters.

Metric	Value
Epoch count	100
Optimizer	Stochastic gradient descent (SGD)
Loss function	Cross-entropy loss
Learning rate	0.001
Model momentum	0.9
Batch size	10

**Table 3:** Performance metrics.

Model	COVID + correct classification (TP)	COVID + wrong classification (FP)	COVID correct classification (FN)	COVID wrong classification (TN)
DenseNet121	47	3	99	1
ResNet18	47	3	97	3
SqueezeNet	44	6	98	2

**Table 4:** Model training comparison.

Model	Time to predict 200 images (s)	Time to predict a single image (s)
DenseNet121	18.85	0.09
ResNet18	10.13	0.05
SqueezeNet	9.90	0.04

**Table 5:** Prediction scores using different metrics.

Model	Accuracy	Sensitivity	*F*1-score
DenseNet121	97.33	94.00	95.92
ResNet18	96.00	94.00	94.00
SqueezeNet	94.67	88.00	91.67

## Data Availability

The data that support the findings of this study are available upon request from the corresponding author.
